# Randomized controlled trial to evaluate screening and brief intervention for drug-using multiethnic emergency and trauma department patients

**DOI:** 10.1186/1940-0640-8-8

**Published:** 2013-04-08

**Authors:** Kimberly Eisenberg, Susan I Woodruff

**Affiliations:** 1 Center for Alcohol and Drug Studies, 6386 Alvarado Ct. Suite 224, San Diego, CA, 92120, USA; 2 San Diego State University, School of Social Work, 6386 Alvarado Ct. Suite 240, San Diego, CA, 92120, USA

**Keywords:** SBI, Emergency room patients, Drug use, Health educator, Curriculum, Training, Process measures

## Abstract

**Background:**

Screening and brief intervention (SBI) is a comprehensive, integrated public health approach to identify and deliver a spectrum of early detection and intervention services for substance use in general medical care settings. Although the SBI approach has shown promise for alcohol use, relatively little is known about its effectiveness for illicit drug use. We are evaluating the SBI approach for drug use using a rigorous randomized controlled trial. The purpose of the report is to describe the overall trial and its programmatic and methodological strengths with a focus on health educator (HE) selection and training. In addition, the baseline characteristics of the recently enrolled multiethnic cohort are described.

**Methods/design:**

A randomized two-group repeated measures design is being used in which drug-related outcomes of an intervention group will be compared with those of an attention-placebo control group. Selection of bicultural paraprofessional HEs—their training in research concepts, comorbid mental health issues, special treatment of marijuana use, and nonscripted enhanced motivational interviewing as well as their ongoing monitoring and evaluation—are among the features described. The HEs enrolled, consented, and conducted an intervention among 700 illicit drug users in two large hospital emergency departments/trauma units. To be eligible, a participant needed to be an adult (age ≥18 years), an English or Spanish speaker, awake and able to give consent, and reachable by telephone to schedule a six-month follow-up interview.

**Discussion:**

A comprehensive HE training protocol combined with rigorous, ongoing process measurement resulted in skill mastery in many areas and a successful participant recruitment period. Strengths and limitations of the study protocol are discussed as well as the characteristics of those recruited. This trial will be among the first to provide information about the effectiveness of SBI for illicit drug use. Outcome analysis has not yet been completed, but demonstrated programming and design successes have implications for future research and service delivery.

**Trial registration:**

http://NCT01683227

## Background

Screening and brief intervention (SBI) is a comprehensive, integrated public health approach to identify and deliver a spectrum of early detection and intervention services for substance use in general medical care settings. These settings, such as emergency departments, offer a potential “teachable moment,” because patients may have perceptions of vulnerability about their health, and, therefore, be particularly receptive to screening and counseling irrespective of the health complaint that led them to seek medical care [1]. There is mounting scientific evidence suggesting SBI is effective at reducing alcohol use at varying levels of severity in a myriad of health care settings including primary care, emergency departments, and trauma centers [2-4]. However, because patients may change on their own after a health care visit, we best rely on randomized trials to test the efficacy of SBI.

Although the SBI approach has shown promise for alcohol use, relatively little is known about its efficacy for adult illicit drug use [5]. An international study reported that, in most countries, brief intervention delivered in outpatient health care settings was associated with very small reductions in illicit substance use as measured by self-report at three months [6]. The exception in this study was the United States, in which participants *and* controls changed in a positive direction; however, the controls changed more than participants. In a six-state study by Madras and colleagues [7], a 68% reduction in self-reported illicit drug use was found among participants exposed to SBI, although the study lacked a control group. A randomized controlled trial of out-of-treatment cocaine and heroin users screened by peer interventionists during urgent care visits reported a salutary effect of SBI on drug use [8]. However, with the exception of this study, methodological issues, such as lack of biological confirmation of drug use, short follow-up periods, and lack of control groups, limit conclusions drawn from existing research on intervention effectiveness.

Alcohol SBI is quickly becoming a recommended best practice in a variety of settings; however, convincing evidence from rigorous studies is needed before the approach is adopted for illegal drug use [5]. The present randomized controlled trial is assessing the effectiveness of the SBI approach for drug use among a large sample of ethnically diverse patients visiting emergency departments at two large urban hospitals. The trial has several unique intervention aspects and methodological strengths: (a) recruitment of polydrug users and patients with the full range of drug use severity allows for a pragmatic “real world” test of SBI, (b) use of an attention-placebo control group, (c) biologically confirmed (hair sample) testing for abstinence at follow-up, and (d) employment of paraprofessional bicultural health educators (HEs) to recruit and deliver the interventions. To our knowledge, no specific training curriculum has been developed for an SBI drug use intervention study that adequately addresses the elements of recruiting participants into a randomized controlled trial, the mental health challenges and multicultural nature of the population, or the prevalence of quasilegal marijuana use in San Diego, CA. The purpose of this report is to describe the overall trial and its programmatic and methodological strengths with a focus on HE selection and training. In addition, the baseline characteristics of the recently enrolled multiethnic cohort are described.

## Methods

### Design and procedures

Life Shift/Shift Gears is a NIDA-funded study to evaluate the effectiveness of SBI for drug use among patients visiting large urban emergency departments/trauma units for various reasons not necessarily related to illicit drug use. A randomized, two-group, repeated measures design is being used in which drug-related outcomes of an intervention group will be compared with those of an attention-placebo control group.

Participants were recruited from two large hospital emergency departments and trauma centers. Using a scripted interview guide, the HE explained to potential participants that they might be eligible for a health study and asked their permission to prescreen them privately. Exclusion criteria were age <18 years, severely altered mental status, being physically incapable of participating due to severe illness or injury, being without any phone number where they could be reached for follow-up, and being unable to speak English or Spanish. Further eligibility was based on responses to two prescreen items that assessed recent alcohol and illicit drug use. The questions were, “How many times in the past 30 days have you used alcohol?” and “How many times in the past 30 days have you used any nonprescribed drugs, such as marijuana, cocaine, or club drugs?” Patients were not expected to report using drugs when in fact they were not using them; therefore, hair samples to confirm self-reported drug use were not collected at baseline. Along with the alcohol and drug prescreen questions, “distracter” questions assessing nutrition, exercise, and driving/traffic safety were asked so that potential participants did not guess the primary purpose of the study. Those answering “no” to the current use of alcohol and drug questions were verbally reinforced and thanked for their interest. Those reporting alcohol use only were given a brochure that described safe drinking limits and provided internet and community resources for alcohol problems, and thanked for their interest. Those reporting illicit drug use were further considered for inclusion. A considerable number of drug users were also users of alcohol, and in fact, depending upon its severity, alcohol use may pose more harm to the individual than drug use. We were interested in enrolling patients whose illicit drug use severity was equal to or exceeded their alcohol use severity, yet ethical considerations demanded that we appropriately address the more severe problem. Both drug and alcohol risk levels were determined for this set of patients using two validated and widely used brief screens available in both English and Spanish interview formats: the Alcohol Use Disorders Identification Test (AUDIT) [9] and the Drug Abuse Screening Test (DAST-10) [10]. Health educators computed AUDIT and the DAST scores immediately and applied standard cut-points to determine risk categories. The AUDIT alcohol risk categories were based on Babor et al. [9] and included no/low risk (score of 0–7), at risk (score of 8–15), high risk (score of 16–19), and severe risk (score of 20–40). The DAST-10 categories were based on those of the test’s developer [10] and included no/low risk (score of 0), at risk (score of 1–2), high risk (score of 3–8), and severe risk (score of 9–10). Patients whose alcohol use risk category exceeded their drug use risk category were given a brochure that described safe drinking limits and were provided with information on internet and community resources for alcohol problems. Those patients whose drug use risk category was equal to or higher than their alcohol risk category were considered eligible and were then asked if they would like more information about the study. Potential participants were offered $5 on the day of enrollment and baseline assessment and $20 after completion of the six-month follow-up interview.

If an individual screened eligible and consented, he or she was then randomly assigned to one of two conditions. The intervention group (Life Shift) received SBI drug intervention matched to their DAST risk level and drug of choice as identified by participant self-report. To assess the “true” effect of the SBI intervention (i.e., minus the effects of attention), a control group received the same quantity of intervention in the area of driving and traffic safety (Shift Gears) that did not focus on drugged driving. Baseline and six-month follow-up measures were collected for all participants and included standardized subjective drug use measures from the Addiction Severity Index-Lite (ASI-Lite) [11] and other measures presumed to be targeted by the intervention (e.g., functional status, self-efficacy). At face-to-face follow-up conducted by separate measurement technicians, a hair sample in a length corresponding to past-month exposure was tested for the presence or absence of four major categories of illicit drugs (i.e., amphetamines and other psycho-stimulants, cocaine and its metabolites, opiates, and cannabinoids) to increase the accuracy of subjective abstinence reports. In addition, driving and traffic-safety control items were collected to test the result of the experimental manipulation, with the attention-placebo control group expected to have greater pre/post change on these measures than the SBI intervention group.

### Overview of the health educator model

Life Shift/Shift Gears was modeled after the California Screening, Brief Intervention, and Referral to Treatment (CASBIRT) program, a service that provides universal screening and intervention services to patients in 12 hospital emergency departments and trauma centers throughout San Diego County [12]. Although SBI services can be delivered by different types of personnel, including medical staff, trained therapists, and paraprofessionals [13,14], Life Shift/Shift Gears used paraprofessional HEs. For many SBI programs, well-trained HEs provide high quality, cost-effective counseling services [15]. Although there are likely benefits for having SBI activities delivered by physicians or other health care providers, time constraints and comfort levels may make this option unfeasible [14]. Specially trained paraprofessionals, such as the HEs in this study, have frequently been used as an alternative, with favorable outcomes with regard to costs [16] and patient satisfaction [17]. In addition, the use of peer HEs who screen patients for substance use outside the medical encounter might serve to reduce actual or perceived social stigma [18].

### Health educator selection and hiring

The Life Shift/Shift Gears HE selection and hiring process focused on finding candidates with two core competencies. The first was several years of experience in human services and a commensurate respectful, nonjudgmental attitude toward illicit drug users. The second was written and spoken fluency in English and Spanish in order to accommodate the cultural and linguistic needs of the population of San Diego.

The applicant pool included a number of individuals with SBI experience. Although prior experience is normally advantageous, in this case it presented a challenge: some candidates balked when they realized the control group participants would not receive an intervention for their illicit drug use, but rather would receive an intervention related to driving and traffic safety. For candidates accustomed to providing brief interventions to any patient who met the appropriate risk level criteria, the prospect of delivering the control group intervention was daunting. Behavioral interviewing questions were utilized to screen out candidates who indicated they had personal conflicts with the control group component of the research study.

Three HEs were hired for Life Shift/Shift Gears, two of whom had previous SBI experience and college level education in health and human services. The third HE was new to SBI but had recently earned a Master of Public Health and possessed research experience. All three were bilingual/bicultural and embodied the respectful, empathic attitude essential for effective SBI delivery.

### Health educator training protocol

HE training, the content of which was organized into an interactive manual, was conducted over a one-month period facilitated by project staff. Four core modules were included: Research Methods, Illicit Drug Use, Mental Health, and Motivational Interviewing. Upon completion of the modules, the HEs spent a full week engaged in extensive role-play and practice sessions followed by several days of evaluation and credentialing.

#### Module 1: research methods

The first training module placed heavy emphasis on the basics of human-subjects research with a focus on experimental design and the purpose of randomized controlled trials. Ethics in human-subjects research and the role of Institutional Review Boards were explored in depth, especially with regard to informed consent. Unlike SBI service programs, research requires prospective participants to complete the consent process prior to enrollment. In order to successfully complete the research module and begin participant recruitment, the HEs completed our university’s IRB tutorial and certification process. All three successfully passed the test on their first attempt.

The final element of this module was an overview of the core components of the study, including the hypothesis/research question, the experimental design, the project timeline, implementation flowcharts, and explanations of assessment tools and measurements. Although the HEs understood their role was limited to participant recruitment, assessment, and intervention activities, it was important for them to have a holistic view of how all the study components interrelated. It was expected that the more the HEs understood the study as a whole, the more engaged and effective they would be in their positions.

It was particularly important for the HEs to appreciate the value of the attention-placebo control group. Hired in part for their empathy for the study’s participants, the control group intervention was somewhat antithetical to their instincts. However, they were able to accept its value once they understood that the purpose of the control group was to determine if reductions in drug use over time were actually due to SBI, or if attention alone could cause behavioral change. The Research Methods curriculum allowed the HEs to move past their personal feelings about delivering the control group intervention and endorse the experimental design of the study, as evidenced by quality assurance monitoring (described in the Process Measurements section of this paper).

#### Module 2: illicit drug use

This module included an overview of the biological mechanisms by which drugs of abuse act, their potential impacts on low risk users, as well as the diagnoses of abuse and dependence [19]. It also covered common drugs of abuse, their street names, routes of administration, short- and long-term effects, and consequences of ongoing use, including use at low risk levels or by individuals who do not meet diagnostic criteria for abuse or dependence. According to data from CASBIRT, the most common drugs of abuse in San Diego are marijuana, methamphetamine, and cocaine, so particular attention was paid to these substances. The HEs were taught about common treatment modalities, including detoxification and therapeutic communities (inpatient and outpatient). They were provided with lists of local resources to make appropriate referrals, as referral to treatment was routinely offered to experimental group participants who fell into higher categories.

The most nuanced elements of this module were the two areas that relied heavily on HE use of critical thinking skills to make well-reasoned situational judgments. The first such area was a harm reduction approach. Although abstinence is the best option with respect to illicit drug use, it is often unrealistic to expect that an individual will be willing and able to immediately achieve abstinence, even following a powerful brief intervention conducted at a teachable moment. For this reason, HEs could employ a harm reduction approach for participants who were not yet ready to consider abstinence [7]. By training the HEs about the positive outcomes associated with meaningful reductions in illicit drug use, they became prepared to negotiate interim reductions while still ultimately advocating for abstinence.

The other unique element of this SBI training was endemic to San Diego: the prevalence of marijuana use. Although marijuana remains illegal at the federal level, liberal legislation at the state level [20] combined with permissive social norms (e.g., prevalence of dispensaries and “smoke shops” in urban and suburban areas and easily obtained medical-marijuana cards) create a culture in which many residents view marijuana use as a benign, socially acceptable activity. Our data collection tool captured whether or not marijuana users were using at the advice of a medical professional, as this is a common phenomenon in San Diego; however, though HEs were respectful of participants’ claims of medicinal use, they treated them with the same protocol as all participants. Because of the popular perception that marijuana use rarely results in medical, social, or legal consequences, HEs needed to be able to engage participants in respectful dialogue about their views regarding perceived benefits and minimal risks while still counseling them about the detrimental health and social consequences associated with ongoing use.

Training the HEs to approach marijuana in a way that was appropriate and respectful of cultural norms while remaining in harmony with the principles of brief intervention and the known risks of marijuana use was accomplished in several steps. The first step was to develop an understanding of the effects of marijuana, including the physical, mental, and psychological impacts of long-term use [21]. Next, the HEs read information about responsible use [22] and the veracity of claims of harmfulness [23]. Finally, once they possessed an information base of medically accepted standards as well as political/cultural rhetoric, HEs role-played scenarios in which they debated the merits of use and nonuse and were challenged to consider viewpoints that differed from their own. This process helped the HEs prepare to handle complex real-world discussions about participants’ marijuana use and to use motivational interviewing (MI) techniques to steer participants toward healthy behavioral change.

#### Module 3: mental health

Data from CASBIRT indicated that co-occurring mental health disorders were common among illicit drug users who use emergency and trauma services at urban San Diego hospitals. To understand how to best work with this patient population, the mental health training curriculum began with overviews of the mental illnesses HEs were most likely to encounter: Axis I disorders, including mood, anxiety, and psychotic disorders; Axis II personality disorders; and co-occurring disorders, since many patients who use illicit drugs simultaneously suffer from mental health challenges [24]. Prominent signs and symptoms were explained to help the HEs identify these disorders based on the behaviors and affect of prospective participants. The mental health module was designed in part to reduce HE anxiety about working with these patients while simultaneously decreasing stigma, which is an ongoing challenge for people living with mental illness [25]. There was also a section dispelling the connection between violence and mental illness, a popular cultural misconception with minimal basis in reality [26].

The most critical element of the mental health module was discussion of which types of patients were suitable for enrollment. It was essential that the HEs be able to apply critical thinking skills when recruiting mildly symptomatic individuals into the study. Since inclusion criteria only required that a participant be awake, alert, and able to engage in the interview, patients with mild to moderate mental health challenges often met these criteria and were considered appropriate to be invited to participate. Patients who were floridly psychotic or otherwise symptomatic to the point of distraction were not considered eligible for participation.

#### Module 4: motivational interviewing

Motivational interviewing is the counseling style most commonly associated with SBI [9] and the means by which HEs attempted to elicit participants’ internal motivation and encourage healthy behavioral change. Life Shift/Shift Gears was designed to deliver SBI services with an enhanced brief MI component. Unlike other SBI programs that rely heavily on a script, this study sought to instill the HEs with robust MI competencies.

Training in MI consisted of two weeks of presentations by MI experts, written exercises, videos, and role-playing practiced with each other and with project staff and volunteers. Role-playing offered an opportunity for the HEs to refine their skills in both English and Spanish with a variety of individuals, many of whom they had never met before, and all of whom attempted to approximate the diverse participants the HEs would encounter in the field.

The HEs practiced MI-informed delivery of the brief intervention using informational brochures developed to guide the brief interventions for the experimental and control groups. These brochures, available in English and Spanish, served as a template for service delivery and were used to supplement HE skill development. Brochures for participants in the experimental group were produced for marijuana, methamphetamine, cocaine, and general drug use (for participants whose primary drug of abuse was not one of the first three). In addition to information about the risks of illicit drug use, the brochures included decisional balance grids and personal change plans for the HE to complete with interested participants as an element of the brief intervention. Similarly formatted brochures were used for patients in the control condition. These included information about risky driving and traffic safety behaviors.

#### Evaluation and credentialing

Upon completion of the training modules, the HEs were evaluated on their mastery of each content area. Written tests were administered, and each HE was observed while conducting mock interviews in both English and Spanish. Performance was scored using a highly customized observational checklist that included each task the HE was required to complete, from the initial greeting of a participant through screening, consent, assessment, and intervention. After successful completion of the tests and mock interviews, the HEs began participant recruitment.

### Interventions

The Life Shift drug intervention and the Shift Gears driving/traffic safety intervention were manual-driven, designed to be of the same duration (10–30 minutes), and available in English and Spanish. Both interventions had core SBI elements that all participants received as well as adaptive elements that allowed the HE to tailor the program based on the patient’s needs and risk level. Both interventions began with the HE providing the participant’s risk level in a nonjudgmental manner using a brochure to help communicate the short- and long-term health, social, and legal effects. For the Life Shift intervention, drug-specific brochures were used when marijuana, cocaine, and methamphetamine were the drugs of choice; otherwise, a general drug use brochure was used. For the Shift Gears intervention, a single brochure was used that focused on issues such as nonattention, road rage, and texting while driving. The patient’s motivation or readiness to change was then assessed using a four-point scale. The readiness information was used to guide additional discussion about the patient’s drug use (or risky driving/traffic behaviors). If the participant was ready to change, the HE discussed possible behavioral changes the patient could incorporate into his/her life. If the participant was not ready to change, the HE helped the patient weigh the pros and cons of his or her present behavior and the pros and cons of change. Together, the HE and participant made a plan specific to the participant based on the discussion and recorded ideas on the worksheet on the back of the brochure.

### Participant recruitment and follow-up

**Figure 1:**
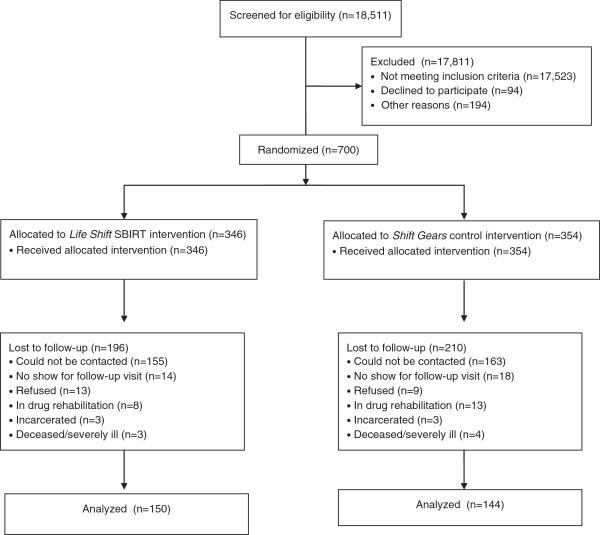
CONSORT diagram showing the flow of patients through the trial.

Participant recruitment was conducted from April 2010 through June 2011, with a total of 700 subjects enrolled. Modifications to the experimental protocol were minimal. Figure 1 presents a CONSORT diagram of the flow of patients through the trial. Over 18,000 patients were approached and screened for their eligibility and interest in participating. The great majority of those excluded did not meet the eligibility criteria (e.g., their alcohol use risk exceeded their drug use risk). Of those who were eligible to participate, 29% were excluded, with 9.5% refusing and 19.5% excluded for other reasons (e.g., being interrupted for a medical procedure, becoming too sick or too sleepy to continue). The six-month follow-up rate was 43% with no difference in reasons for loss by condition.

### Outcome measures and analyses

Mixed model analysis of variance (ANOVA) procedures within a general linear model framework will be conducted on the key self-reported outcomes measured by the ASI-Lite. The ASI-Lite composite scores of recent drug use and related medical problems, psychiatric problems, and alcohol use will be analyzed along with health-care utilization outcomes to assess differential change by condition. Because of potential bias introduced by a less-than-ideal response rate, two sets of analyses for the self-reported data will be conducted: complete-case analyses of 294 participants with longitudinal data and intention-to-treat analyses using multiple-imputation data to deal with loss to follow-up (N = 700). With multiple imputation, missing follow-up values are replaced by a series of plausible values rather than by a single value [27-30]. For each outcome, we will use multiple imputation with a chained equation approach to create five imputed data sets and pool these results. Imputed outcome values will be predicted from baseline drug and alcohol use variables, gender, age, income, and drug avoidance self-efficacy. In addition to changes in composite scores, the ASI-Lite drug use scores will be dichotomized into a past 30-day drug use abstinence variable (yes/no) measured at the six-month follow-up. Results of hair analysis will be used to verify abstinence reports. When hair analysis is positive for drug use and self-report is negative for drug use, the participant will be considered nonabstinent.

### Participant baseline characteristics

Table 1 presents the sociodemographic and baseline characteristics of patients enrolled in the study, both overall and by condition. The similarity of participants in the two conditions indicates the success of the random assignment. About three-fourths of the participants were men. The average age was in the mid-thirties, although participants were well-distributed across the age ranges. The sample was ethnically diverse, with one-third being Latino. The great majority of patients were enrolled and received the intervention while admitted to hospital emergency departments (versus those hospitalized in level 1 trauma centers). Severity of drug use varied, with over half of the sample screening “at risk” for drug use, and about 45% at “high” or “severe” risk [10,31]. Marijuana was by far the most commonly used drug (84%) followed by amphetamines (19%). Use of more than one substance (including alcohol) was common, with about half of the participants reporting such use patterns. A measure of self-efficacy for avoiding drugs [32] was “moderate” at baseline (3.2 on a five-point scale ranging from low to high self-efficacy). Acculturation level among the 232 Latino participants [33] was 3.6 on a five-point scale ranging from low acculturation to high acculturation. Although interview instruments and intervention materials were available in Spanish, it was rare for participants to ask to be interviewed in Spanish.

**Table 1:** Characteristics of life shift/shift gears participants

	**Percent or mean (SD)**		
	**Overall**	**Life shift**	**Shift gears**
	**(N = 700)**	**(n = 346)**	**(n = 354)**
Gender (%)			
Male	75.4	76.2	74.6
Female	24.6	23.8	25.4
Age category (%)			
18-20	9.3	10.7	7.9
21-24	13.2	13.6	12.7
25-34	27.1	29.6	24.6
35-44	17.5	16.6	18.4
45-54	22.1	19.5	24.6
55+	10.9	10.1	11.6
Mean age in years	36.9 (13.2)	35.9 (13.3)	37.9 (13.0)
Race/ethnicity (%)			
Hispanic/Latino	33.1	32.5	33.7
African American	37.0	36.5	37.4
White	24.8	26.0	23.6
Other	5.2	5.0	5.3
Patient location (%)			
Emergency Department	83.5	84.3	82.6
Hospitalized for Trauma	16.5	15.7	17.4
DAST drug risk category (%)^a^			
At risk (scores 1–2)	55.1	55.0	55.2
High risk (scores 3–8)	41.1	40.6	41.7
Severe risk (scores 9–10)	3.8	4.4	3.2
Type of drugs used in the past 30 days (%)^b^			
Marijuana	84.4	87.5	81.4
Amphetamines	19.3	17.4	21.2
Cocaine	8.8	7.8	9.7
Heroin	7.8	6.1	9.6
Other opiates	7.4	7.6	7.3
Use of more than 1 substance, including alcohol (%)	49.9	47.9	51.9
Use of more than 1 substance, excluding alcohol (%)	30.4	29.6	31.3
Drug avoidance self-efficacy score (mean)^c^	3.2 (1.2)	3.2 (1.2)	3.3 (1.2)
Acculturation score among Latinos (mean)^d^	3.6 (1.2)	3.6 (1.3)	3.6 (1.2)

^a^ There was no “No risk” category due to the eligibility criterion.

^b^ Prevalence based on ASI-Lite individual drug use items.

^c^ Scores potentially ranged from 1 (low self efficacy) to 5 (high self efficacy).

^d^ Scores potentially ranged from 1 (low acculturated) to 5 (high acculturated).

### Process measurements

Activities of HEs were monitored on an ongoing basis through a variety of process measurements. Daily and weekly reports were designed and used to track the success of participant recruitment efforts. A Daily Recruitment Report (DRR) was submitted via email to project management and included total number of patients screened, number eligible for enrollment, total number enrolled, and reasons that otherwise eligible patients did not complete the enrollment process (e.g., interview interrupted, did not consent). The DRR provided a real-time picture of HE activity and allowed project management to give immediate positive feedback when an HE had a high enrollment day.

A Weekly Participant Recruitment Report (WPRR) was submitted by each HE at the same time each week that contained a greater level of detail about their recruitment efforts and the progress they were making toward overall recruitment goals. The information collected was tallied on a form throughout the week and then compiled into a report and disseminated to all project staff so everyone was apprised of recruitment status on an ongoing basis. The WPRR included the following variables: total patients screened, patients who refused initial screening, eligible participants who dropped out during the assessment, eligible participants who dropped out during the intervention, interrupted/incomplete enrollments, eligible participants who did not consent, eligible participants who did not have contact information, and completed enrollment interviews.

Collection of these process measures allowed project management to evaluate the implementation of the experimental design and make modifications as soon as shortcomings were identified, or if HE productivity began to wane (although it rarely did). For example, having this information in real time from the first day of participant recruitment resulted in immediate adjustments to HE work schedules to ensure recruitment activities were conducted on the days and times that they were most likely to yield results. Since the process measures were shared with the whole team, the information helped to motivate the HEs to engage in friendly competition with their colleagues and keep their individual recruitment numbers high. These measures also helped project management more accurately predict when recruitment goals would be attained, and plan accordingly.

Other process measures were collected as part of the participant interview process and recorded in the data collection instrument. Such variables included the hospital location and whether the participant was an emergency or trauma patient. This knowledge helped illuminate which locations and departments were the most fruitful for recruitment and which environments were best suited for administering SBI. Start time and end times of each intervention were also recorded, which served a dual purpose. First, it identified peak recruitment times, which was helpful for scheduling purposes. Second, it allowed project management to monitor the time HEs spent delivering the intervention. From a quality assurance perspective, it was critical to monitor the time of the brief interventions to ensure they were lasting the intended amount of time and that both the experimental and control groups received interventions of the same average duration.

Quality assurance was the responsibility of the project manager, who possessed a Master of Social Work with a concentration in administration, undergraduate education and experience in chemical dependency counseling, and multiple years of experience as a project manager. Drawing on this background, the project manager shadowed the HEs as they conducted participant enrollment interviews and, utilizing the observational checklist, was able to reinforce effective techniques and offer immediate feedback about areas in need of improvement. While the HEs’ performance generally met or exceeded expectations, there were rare instances in which the project manager observed drift from the experimental design. Most commonly, this occurred when a HE missed an opportunity to delve further into a participant’s behaviors and uncover motivation to change.

To mitigate the natural process of drift from the experimental design, the HEs remained engaged in ongoing training activities for the duration of the recruitment period. They met with the project manager on a regular basis and continued to engage in didactic and experiential MI training. They were also treated as a valuable project resource; when areas in need of improvement were identified, the HEs were actively solicited for feedback and suggestions on what could be done differently. By approaching the HEs in this collaborative manner, the project benefited from their insights while simultaneously strengthening their engagement in their respective roles.

## Discussion

### Strengths

Training personnel to conduct SBI as part of standard medical care requires a comprehensive approach, but training paraprofessional HEs to conduct those same services for a randomized controlled trial requires greater depth and breadth of training. Although Life Shift/Shift Gears had not been completed at the time of publication, the study’s carefully monitored implementation and subsequent recruitment of 700 participants is a testament to the strength of the training curriculum. As observed through a variety of process and quality assurance measures, HEs consistently delivered quality SBI services to participants while adhering to the experimental design.

The customized curriculum allowed the HEs to develop a true appreciation for the experimental design, strong MI skills, and enhanced critical thinking skills about the complex issues of drug use and behavioral change. The extensive role-playing, followed by the evaluation and credentialing process, bolstered HE preparedness to begin participant recruitment. Once recruitment began, monitoring their activities allowed for modifications to the schedule and associated protocol and safeguarded against drift from the experimental design. Regular training kept HE skills current so they were able to deliver high-quality services throughout the duration of the recruitment period. Finally, by treating the HEs as valued, integral members of the team, they were encouraged to contribute at higher levels while concurrently strengthening their dedication to the project.

### Limitations

Due to its pragmatic nature, this study is primarily a test of effectiveness, but the attention-placebo control group has the characteristics of an efficacy trial. One of the challenges in hiring and training personnel was to simultaneously prepare them for a randomized controlled trial and a real-world SBI. As a result, there are limitations to transportability. Most notably, the training period for HEs was a full month, probably longer than can realistically be expected for service programs. However, a large portion of the training was dedicated to research concepts, which accounts for most of the disparity between the training duration of this study and what practitioners could reasonably accommodate. The other issues of transportability are related to funding and the ongoing question of whether SBI services should be delivered by paraprofessionals or trained medical staff. Enthusiasm for SBI services has been building in California in recent years, and the CASBIRT health educator model, upon which this study was built, has been recognized as a realistic alternative in health-care settings where medical personnel are unavailable [34]. Similarly, Bernstein and colleagues [5] found that, regardless of setting, SBI programs tend to migrate to the use of HEs over medical professionals. They also found that, although SBI funding is not guaranteed, as the model continues to result in cost savings, and as new billing codes are activated, it becomes increasingly likely that there will be funds to support these positions.

Although the HEs in this study were successful in their recruitment efforts, and despite concerted cohort maintenance efforts, a considerable number of participants were lost to follow-up, a common problem in studies of drug users [35,36]. Project staff aggressively followed up with participants by phone and mail and made home visits when it was feasible and safe. Study design did not allow for follow-up with participants who were incarcerated or enrolled in inpatient treatment; if it had, it is possible that the follow-up rate would have been higher. A low follow-up rate is of concern because of potential threats to validity, especially to the degree that the characteristics of those lost to follow-up differ by experimental condition. We plan to conduct a thorough analysis to test attrition bias and to conduct outcome analyses that adjust for missing data.

Although it was an advantage to collect hair at the follow-up assessment, hair was not collected at baseline. It is possible that non-drug users could have been enrolled in the study, although reporting of drug use that did not occur is thought to be minimal [37]. We were not able to test for all possible drugs, although participants were tested for the most common drugs of abuse in San Diego County. Like any biological measure, hair samples have shortcomings, and statistical and technical problems related to their use to validate self-reports of drug use have been reported [38]. Adequacy of screening instruments is also a concern; for example, although the DAST is widely used and its psychometric strengths are well-established as a diagnostic tool, there is a lack of information about its validity in an emergency-department setting with a wide range of patients. In addition, although trained in research methods and experimental design, the HEs could have contaminated the groups by providing a drug use intervention to participants in the control group.

Comparing our results to similar studies will be difficult given the lack of randomized controlled trials for drug SBI, differences in the types of drug users targeted, and other important methodological differences. Probably the most similar published study was a randomized trial of brief motivational intervention in clinics for out-of-treatment cocaine and heroin users screened by peer interventionists during an urgent medical visit [8], but that study involved higher risk drug users than those in our sample. We plan to compare our results to the Bernstein et al. study [8] to the degree possible, as well as other studies that may be published soon.

## Conclusions

The successful implementation of this study can be attributed largely to a customized training and development protocol, which led to a high level of skill mastery combined with a flexible approach. The HEs were thoroughly educated in the fundamental concepts, and then taught to think critically about participants’ motivations and perceptions about illicit drug use in order to achieve optimal results. This combination allowed the HEs to successfully recruit, screen, and intervene with large numbers of hospital patients in a way that would not have been possible with a traditional scripted approach. As a result of this innovative design, final results from this study will provide meaningful insight into the effectiveness of SBI for illicit drug use.

## Competing interests

Both authors declare that they have no financial or nonfinancial competing interests.

## Authors’ contributions

SW participated in the conceptualization of the manuscript as well as writing and assisting with statistical analyses. KE wrote a considerable amount of the manuscript and assisted with analysis. Both authors read and approved the final manuscript.
